# Infective Endocarditis Following Aortic Valve Replacement: A Systematic Review

**DOI:** 10.7759/cureus.49048

**Published:** 2023-11-19

**Authors:** Ethan Slouha, Catherine Rood, Venkata Sathya Burle, Hanin Al-Geizi, Lucy A Clunes, Theofanis F Kollias

**Affiliations:** 1 Anatomical Sciences, St. George's University School of Medicine, St. George's, GRD; 2 Pharmacology, St. George's University School of Medicine, St. George's, GRD; 3 Pharmacology, St George's University, St. George's, GRD; 4 Microbiology, Immunology and Pharmacology, St. George's University School of Medicine, St. George's, GRD

**Keywords:** aortic valve replacement, percutaneous aortic valve replacement, transcatheter aortic valve replacement, surgical aortic vavle replacement, infective endocarditis

## Abstract

Aortic valve replacement (AVR) successfully treats aortic valve stenosis and aortic regurgitation from aging or bicuspid aortic valves. The procedure intends to restore the obstructed left ventricular outflow tract (LVOT). AVR can be performed surgically (surgical aortic valve replacement (SAVR); open heart) or via transcatheter (transcatheter aortic valve replacement (TAVR)), typically done through a femoral approach as a minimally invasive procedure, allowing for quicker recovery and reduced hospital stays. AVR has many complications, including life-threatening ones, such as infective endocarditis (IE), retarding the recovery process and increasing mortality following surgery. IE is an uncommon and deadly condition that involves multiple organ systems and is caused by bacteremia stemming from a microorganism that enters the bloodstream. Many manifestations are involved in the development of IE, such as fevers, flu-like symptoms, splinter hemorrhages, Osler nodes, abscesses, and vegetations found on the valves at the leaflets. Vegetations and abscesses tend to create further complications, such as stroke and acute kidney injury, as emboli block blood flow, leading to ischemia and damage. This paper aims to evaluate the difference in SAVR- and TAVR-associated IE, as the goal is to elucidate a danger that diminishes the positive effects of either procedure despite its rarity. Studies have been inconclusive in determining whether or not there is a trend, let alone a difference in incident rates. Both procedures share similar risk factors, but SAVR-associated IE is usually caused by *Staphylococcus aureus, *and studies indicate possibly *Enterococcus spp.* in TAVR-associated IE. Incident rates of IE are much higher than they should be, whether or not they differ between procedures, and future research needs to consider the pathways and risk factors that can be used to reduce the occurrence of AVR-associated IE.

## Introduction and background

Infective endocarditis

Infective endocarditis (IE) is a rare condition associated with high morbidity and mortality [1]. It is defined as an infection of the native or prosthetic heart valve or an indwelling cardiac device [2]. It has also been called bacterial endocarditis, but any microorganism can cause IE. The microorganism enters the bloodstream through any access point, usually oral mucosa or wound with bloodstream access [3]. The microorganisms travel through your bloodstream and can start to cumulate at the lining of the heart or valve, typically when there is damage, causing vegetation [3]. *Streptococcus viridans *used to be the most common microorganism that caused IE, which correlates to one of the main origins of where bacteria can enter: the oral mucosa that has been cut [2]. However, *Staphylococcus aureus* has become more prevalent in recent years, possibly through invasive procedures and healthcare contact, between 9% and 29% [2,4].

The current annual incidence is up to 10/100,000 and carries a mortality risk of up to 30% [1]. Patients are usually admitted with symptoms of fever, fatigue, night sweats, appetite, weight loss, acute heart failure, and sometimes embolic phenomena [1]. To help diagnose IE, the modified Duke criteria is used, which requires a mix of major (vegetation, abscess, positive blood culture, etc.) and minor (arterial emboli, IV drug use, fever, etc.) criteria [1]. Vegetations occur around the leaflets of both native and damaged heart valves as there is reduced blood flow due to the abnormality [2]. The treatment of IE has been relatively consistent, ranging between the use of antibiotics and surgical intervention [1]. The types of antibiotics have changed throughout the years due to advancements in pharmacology, and treatment is usually centered around it, with surgery as a last option [1]. While IE can happen with native valves, prosthetic valves and grafts are more likely to be infected due to the actual procedure used and different surfaces for bacteria to create biofilms [2]. IE can occur following aortic valve replacement (AVR) and lead to devastating complications with high mortality due to the advanced age of patients who undergo AVR.

Aortic valve replacement

AVR is the optimal way to treat damaged and diseased aortic valves. Aortic valve stenosis (AS) is a common valvular complication that leads to a left ventricular outflow (LVOT) obstruction [5]. AS has many etiologies, such as congenital bicuspid aortic valve, calcification from aging, and rheumatic diseases [5]. As AS progresses, life-altering symptoms start to occur, such as exertional dyspnea and severe fatigue; this happens 10-20 years following an asymptomatic period [5]. Ultimately, patients can develop syncope, chest pain, and heart failure [5]. AVR is the only definitive treatment for AS, as no pharmacological approaches have proven effective [5]. AVR involves completely removing and replacing the damaged valve with a new valve made of animal tissues or synthetic materials [6]. There are two ways to do an AVR: surgically or via transcatheter (percutaneous).

Surgical aortic valve replacement

One way that AVR can be done is through open heart surgery called surgical aortic valve replacement (SAVR). The patient is typically placed under general anesthesia, and the physician performs a sternotomy and places the patient on a heart-lung bypass machine [6]. After replacing the valve, the surgeon restarts the heart [6]. Recovery from SAVR takes about two to three months, but the patient is discharged about one week following the surgery [6]. Post-surgical complications include wound, lung, bladder, or heart valve infections, blood clots, strokes, arrhythmias, and reduced kidney functions for a few days [6]. Factors, such as medication, may be given following surgery to increase survival longevity, and the choice varies depending on the type of valve, such as blood thinners for mechanical valves [7]. The type of valve used during surgery is decided between the patient and surgeon after the surgeon presents all benefits and risks associated with each. Surgeons now have been looking more into transcatheter aortic valve replacement (TAVR) as this surgery shows comparable and, in some studies, better rates of success and lower complications [8]. However, specific individuals cannot undergo TAVR, such as younger patients with bicuspid aortic stenosis, due to unfavorable valve morphology, mixed valve disease, and coexistent coronary artery disease [8].

Transcatheter aortic valve replacement

Both SAVR and TAVR relieve the symptoms of LVOT obstruction and improve heart function and life expectancy [9]. Like SAVR, TAVR replaces aortic valves that are diseased or not working correctly and replaces them with a new aortic valve [10]. TAVR is a minimally invasive procedure requiring only a tiny cut to the skin to access the bloodstream, typically the femoral artery [10]. Compared to SAVRs, this patient has reduced mortality and associated risks [10]. TAVR can be done under minimal sedation in one hour, which means a decreased hospital stay and a shorter recovery time [10]. Following both SAVR and TAVR, IE can occur at a higher risk than in native valves. This paper aims to evaluate and compare the incidence rate, risk factors, microorganism/etiology, symptoms, treatment, and outcomes of both types of AVR-associated IE. The goal is to highlight events or weaknesses that lead to IE and may be able to improve to try and reduce the development following both procedures.

## Review

Methods

A methodical and intensive literature search was executed using PubMed, ScienceDirect, and ProQuest records from January 1, 2003 to December 31, 2023. The search keywords included "infective endocarditis after surgical aortic valve replacement," "infective endocarditis after transcatheter aortic valve replacement," and "infective endocarditis after aortic valve replacement." The electronic investigation concentrated on peer-reviewed experimental publications on this article's purpose. Publications not written in English and published before 2003 and duplicates were excluded from the screening method. Once publications were gathered, four independent co-authors analyzed the information. The publications gathered in the investigation were analyzed based on their titles, abstracts, study type, and full-text accessibility. The initial analysis of the three records resulted in 49,682 publications. Keyword specifics and the information in the abstract further narrowed down the selected publications. A total of 24 publications were found that aligned with the scope of this paper according to the following criteria:

Inclusion Criteria

The inclusion criteria included publications between 2003 and 2023; conducted on humans; written in English; focused on AVR-, SAVR-, and TAVR-associated IE; observational, cohort, case-control, or experimental; peer-reviewed; and available in full text.

Exclusion Criteria

**Figure 1:**
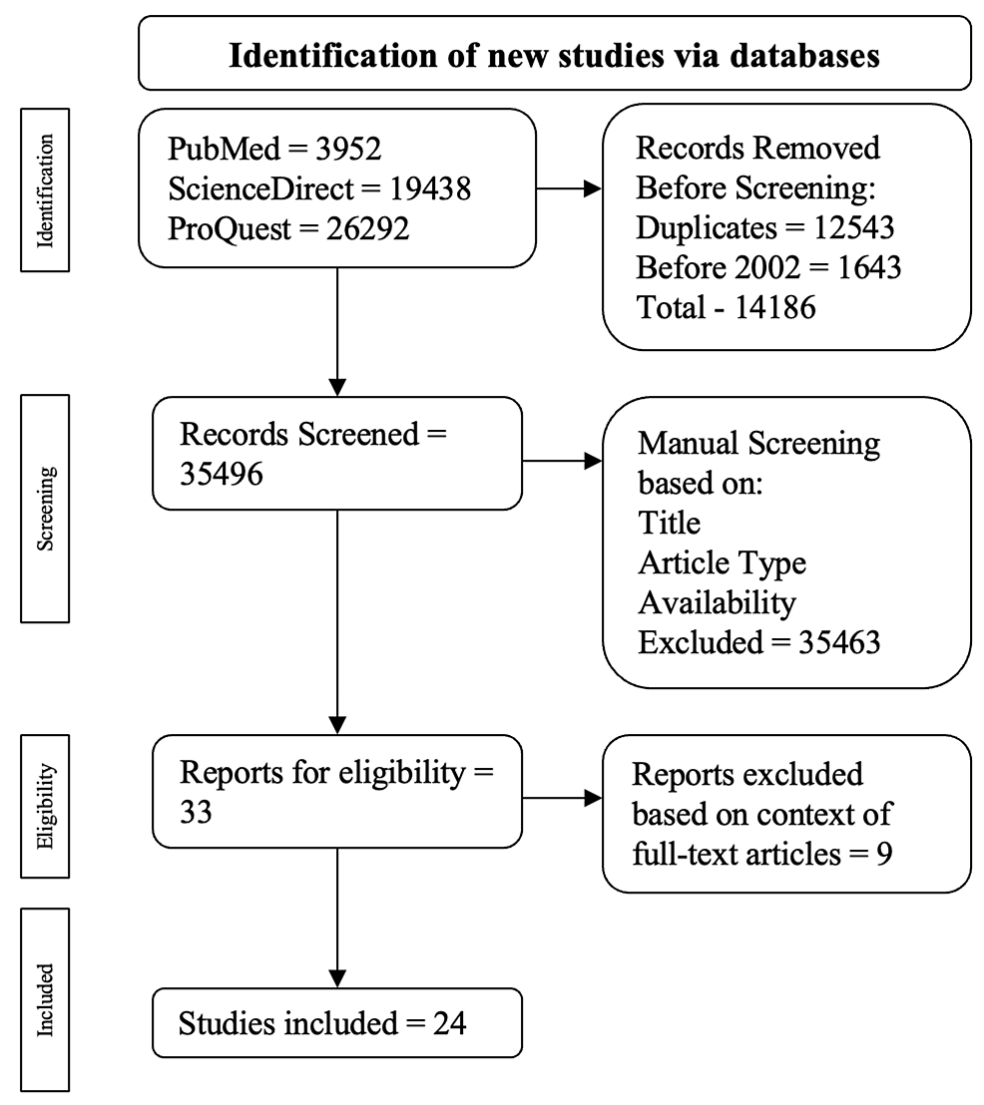
Visual representation of the algorithm used for the inclusion and exclusion criteria. The pathway used for the inclusion and exclusion criteria came from the Preferred Reporting Items for Systematic Reviews and Meta-Analyses (PRISMA) review. Citation: [11]

The exclusion criteria ruled out narrative reviews, meta-analyses, and case series/reports. All non-full-text and duplicate publications were excluded as well. This publication's inclusion and exclusion progression is drawn out in Figure 1.

Bias

**Figure 2:**
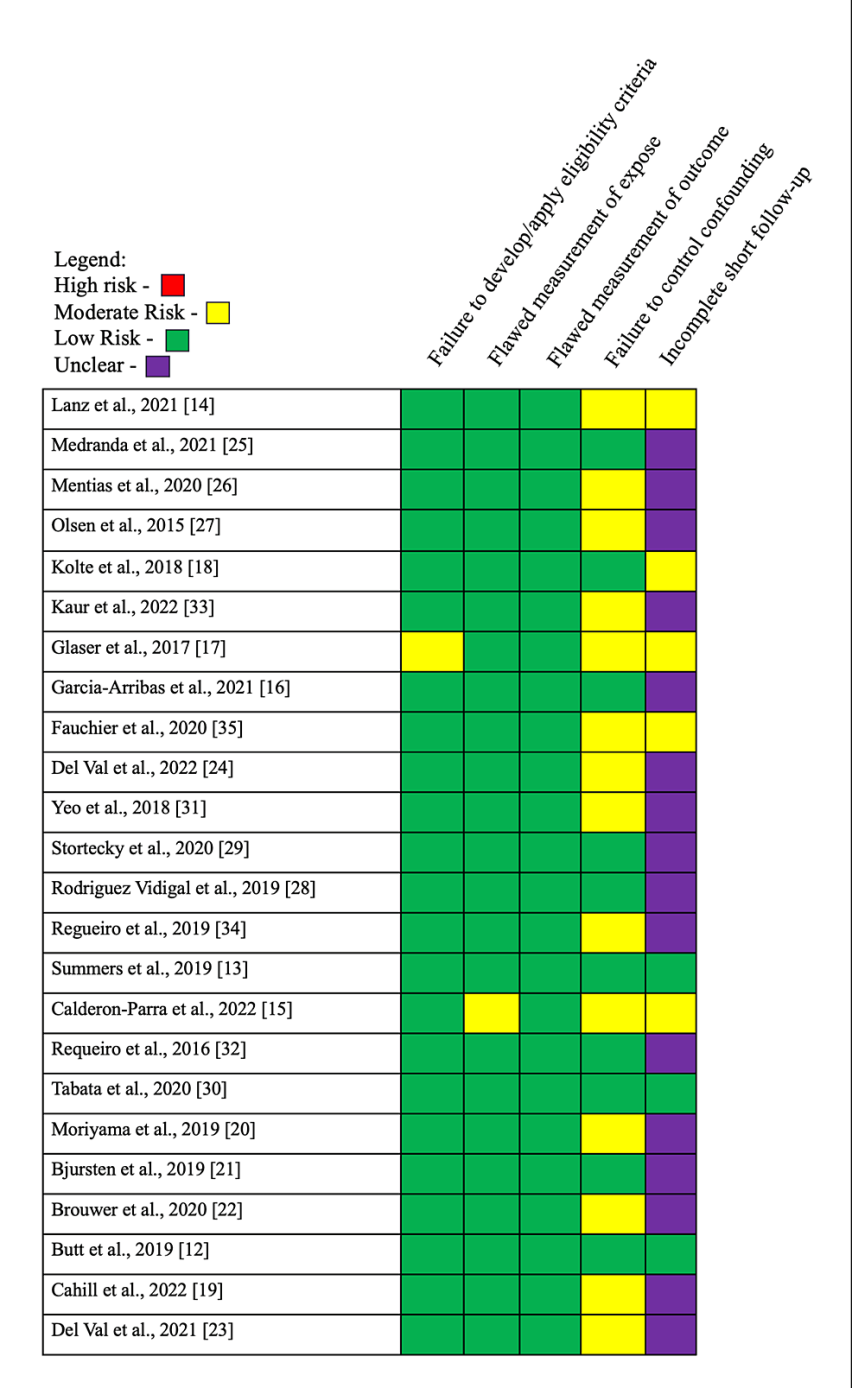
Bias risk assessment per GRADE recommendation GRADE: grading of recommendation, assessments, development, and evaluation

All publications were viewed for bias; most articles focused on one type of procedure. The bias in the papers is minimal, and all methods are explained appropriately. Concerning each article, a moderate GRADE (grading of recommendation, assessments, development, and evaluation) scale rating was given. The GRADE tool was used to assess the risk of bias in individual papers, which weighs flaws like indirectness, imprecision, and publications. The process of GRADE evaluation is depicted in Figure 2.

Results

A total of 49,682 publications were found: 3,952 were from PubMed, 19,438 were from ScienceDirect, and 26,292 were from ProQuest. Among the exclusions, 12,543 were duplicate publications, and 1,643 were published before 2003. This resulted in 14,186 publications being excluded during the automatic screening process, leading to 35,496 publications for manual screening. Publications were manually assessed based on the title, abstract, study type, and full-text availability, resulting in 33 articles being checked for eligibility by a full-text analysis. Ultimately, 24 articles were used.

IE is an uncommon occurrence following AVR, which is observed when you compare the numbers of patients who developed IE versus those who did not. Whether there is a variation between TAVR- and SAVR-associated IE's incident rate is inconclusive; however, more studies observed no significant differences in IE development following either procedure. Risk factors did not vary between the procedures as the male sex and previous cardiovascular conditions both caused an increased risk of developing IE. Common microorganisms did go between procedures, with *S. aureus* being the most common in SAVR-associated IE and an inconclusive observance of microorganisms in TAVR-associated IE. However, more studies did observe that *Enterococcus* spp. was more common than other organisms. Manifestations were mostly similar, except patients with TAVR-associated IE developed fewer fevers, and abscess formation may be more frequent in SAVR-associated IE. The treatment of IE procedures varied, with patients with SAVR-associated IE having slightly more extended hospital stays and more likely to undergo surgical therapy than those with TAVR. Overall, there were no statistical differences in mortality, but patients with TAVR-associated IE may have a higher risk of in-hospital mortality. Table 1 displays information on each article used to gather the information used in this paper.

Discussion

Incidence

When evaluating the differences in AVR-associated IE between procedures, it is essential to consider the overall statistics for AVR-associated IE. The incidence of AVR-associated IE varies across studies from 1.25% to 4.7% but remained small overall due to the relative rarity of IE [12,13]. The incidence rate of AVR-associated IE was 5.06/1000 person-years [13]. Another study found that the cumulative incidence of IE five years following AVR decreased by 1.28% [14]. Summers et al. observed that patients developed IE within a median of 0.87 years following AVR [13].

When focusing on SAVR, the average percent of patients who developed IE between several studies was 2.18% but ranged from 0.95% to 4.9% [12-18]. The average incidence rate of IE was 5.7 per 1000 person-years; however, two studies found rates as high as 12 and 25 per 1000 person-years [12-14,17,19,20]. The cumulative one-year risk of developing IE following SAVR was 1.8% in one study, while the cumulative five-year risk ranged from 1.58% to 5.1%, with the average being 3.03% [12,14,19]. Kolte et al. observed that the incidence of early IE (within one year of procedure) was 25 per 1000 person-years [18]. The day IE is diagnosed following SAVR is important to consider as it sets a time frame that can elucidate possible ways IE enters the system. The average days between the procedure and IE was 1,540 days with a median of 625 days [12]. The average age of patients who developed IE was 67.7 years [16]. There are different types of valves placed in SAVR, and one study observed that bioprosthetic and mechanical valves have similar incident rates [18].

In comparison, TAVR-associated IE occurred at an average of 2.01%, with studies ranging from 0.3% to 5.5% of patients [12-15,18,19,21-31]. One study with a development rate of 5.5% admitted that their incidence of TAVR-associated IE was more significant than that observed in a multicenter series [28]. Overall, the incidence of developing IE after each year dropped, with one study reporting an incidence of only 0.52% 10 years later [21]. The average incidence rate of IE between studies was 9.8 per 1000 person-years, with data spanning from 0.14 to 24.7 per 1000 person-years [12,14,19,20,22,29]. Meanwhile, the cumulative incidence of IE after one year was 1.5%, and the five-year average was 4.2%, with a mode of 5.8% [12,14,29]. Stortecky et al. observed that the development of IE occurred more often in the early peri-procedural period at 2.59%, whereas in the delayed early period, it was 0.72% [29]. There is a split on the change in incidence rates of IE throughout the past few years; one study stated that there have been no changes from cases before 2014 and after 2013, but another has found a steady decline [23,26].

An important observation available in studies assessing TAVR-associated IE is splitting IE into early (<one year) and late (>one year); however, the results varied greatly. The incidence of early IE ranged from 0.9% up to 56.3% with an average of 22.4%, whereas the incidence of late IE ranged from 2.8% up to 50.5% with an average of 40.1% [19,21,22,25-27,30,32]. These results contradict a study that found that the incidence rates at the one-year follow-up for both early and late IE were 1.48 and 0.4 per 100 person-years, respectively [29]. Kaur et al. observed that the incidence of early and late onset of TAVR was identical, with most IE occurring at an immediate time at 60% [33]. One study assessed the trends in the diagnosis of early IE and found a decreasing trend [23].

As observed in evaluating SAVR-associated IE publications, TAVR-associated publications compared the incident rate between TAVR valves and procedures. In assessing the approaches for TAVR, one study observed that 55.7% of patients with IE had their TAVR via a catheterization lab, and 42.8% had their TAVR via a hybrid operation [29]. Another study reported no difference in the incidence rates of IE between transapical and endovascular TAVRs [18]. Regueiro et al. observed that 53.1% of patients who developed IE had a self-expanding valve and an incidence of 0.95% one year following TAVR. While 46.9% of patients who developed IE had a balloon-expandable valve with an incidence of 1.25% one year after the procedure [34]. According to one study, IE has significantly decreased throughout generations of balloon-expandable valves [12]. Still, Medranda et al.’s study found that 100% of the patients who developed IE had a balloon-expandable SAPIEN 3 THV [25].

The time a patient acquired TAVR-associated IE is also important to consider, and on average, it was 89 days, with a median time to diagnosis at 202 days [12,18,23,28,29]. Regueiro et al., however, observed that the median time between TAVR and initial symptoms was up to 5.3 months. They also evaluated differences in time to diagnosis between the self-expanding and balloon-expandable valves. They found no difference in the time it took to diagnose IE [32]. Del Val et al. reported that etiological differences contribute to the time IE is diagnosed following TAVR and found that patients infected with *S. aureus *were diagnosed an average of 1.6 months before non-*S. aureus*-infected patients [24]. One analysis on TAVR studies showed the age at which a person tended to develop IE, and the average age of patients who developed IE was 77.25 years old between the studies [18,22,25,33].

Another factor evaluated was who was more likely to develop TAVR-associated IE. These patients were more likely to be men; had a history of diabetes; had higher EuroSCOREs; had a history of neoplasia, chronic renal failure, and chronic lung disease; had a BMI average of 33.1 kg/m^2^; and likely had a transcatheter prosthesis that was mechanically expandable [22,28,29,32,33]. Most studies agreed that patients who had developed IE were at least 79.4 years old, but one study by Mentias et al. observed that their patients tended to be older only in the development of late IE [18,19,26,29].

The studies comparing SAVR- and TAVR-associated IE observed different outcomes in the incidence rate. Four studies found no statistically significant difference between the incidence of IE between groups [12,13,18,20,35]. Caldero-Parra et al., however, found that 4.65% of patients who underwent TAVR developed IE compared to 1.41% of patients who underwent SAVR, and the difference was significant. They also observed that early IE was more frequent in patients who underwent TAVR at 3.2% compared to SAVR [15]. Alternatively, another study found that the incidence of IE following TAVR was significantly lower than those who underwent SAVR, up to 31% [19]. The time a patient acquired TAVR- vs. SAVR-associated IE was similar with patients matched concerning demographics and risk factors [18]. However, concerning patient types, patients who underwent TAVR were more likely to be older and had a more significant burden of comorbidities than patients who underwent SAVR [12].

Risk Factors

Risk factors concerning overall AVR-associated IE found higher rates in patients with severe pulmonary disease, prior cancer, pulmonary hypertension, liver cirrhosis, chest radiation, and higher BMIs [13]. Vascular access-site infection, deep sternal wound infection, and male sex were also positively associated with IE [20]. There is a slight discrepancy on whether or not the length of the procedure increased the risk of IE, as one study found no difference, and another observed that even a slightly longer approach increased the risk of developing IE [13,14].

Risk factors increasing the risk of developing SAVR-associated IE included obesity, alcohol abuse, use of a cardiac implant device, a history of diabetes, atrial fibrillation, anemia, and male sex [12,16,35]. Glaser et al. observed that patients who were given bioprosthetic valves were at an increased risk of developing IE compared to those who were given mechanical valves. They determined that bioprosthetic valves were more susceptible to deterioration and bacterial infections [17]. Garcia-Arribas et al. observed that the risk factor of diabetes was higher in patients with bioprosthetic valves than mechanical valves [16].

Regarding TAVR-associated IE, the most commonly identified risk factors were male sex, younger age at TAVR, advanced heart failure, aortic regurgitation, history of diabetes, end-stage renal disease, liver disease, lung disease, repeat TAVR, prior endocarditis, lower logistic EuroSCORE, history of malignancy, history of drug abuse, sepsis, and fluid/electrolyte disorder [12,15,18,19,21,24,26,30-32]. Other factors, such as whether there was the implantation of a balloon-expandable or mechanically-expandable vale, low implantation position, implantation of more than one valve, and moderate paravalvular leakage, were also associated with IE [19,27,30]. Del Val et al. compared etiological differences and found that self-expanding valves showed an increased risk of early *S. aureus* IE in 59.4% of patients [24]. Regarding *S. aureus*, those without *S. aureus*-caused IE were found to have a BMI of more than 27.2 kg/m^2^ as a significant risk factor [24]. One study found that general anesthesia was more common in patients with IE, but no other research confirmed this [19]. Other factors not regarding the patient’s comorbidities or actual procedures were considered in one study, which found a statistically significant increased chance of developing TAVR-associated IE if the hospitals had smaller hospital bed sizes and lower yearly volumes of procedures [31].

Between most of the articles mentioned and several that compared SAVR to TAVR IE cases, no significant difference was found regarding the risk of IE [12,15,18,20,35]. Male sex was the most common risk factor across all studies, indicating an increased likelihood of developing IE following SAVR and TAVR. In the comparison articles, specifically, a younger population, those with liver disease, those with heart failure symptoms, those who had diabetes, and those who had a cardiac implantable electronic device were found to have an increased risk of IE following both procedures [12,14,15,35].

Etiology

It is essential to consider the overall AVR-associated IE’s etiology, and the most common routes of IE were recent dental work and recent invasive procedures [25]. The most common microorganisms observed to cause IE were *S. aureus, Streptococcus*, and *Enterococcus spp*., with rates up to 30.4%, 29.9%, and 20.5%, respectively, and *S. aureus* is the most common cause [14,16,18,35]. Coagulase-negative *Staphylococci *also led to IE but was only mentioned in one study [14].

The etiology of SAVR-associated IE is most commonly community-acquired IE at up to 78.4% in one study [16]. The most common microorganism to cause IE was found to be *S. aureus*; however, patients with IE stemming from composite valve grafts were caused mainly by *Streptococcus viridans* [13,15,16]. Lanz et al., however, observed that the most common microorganism that led to IE, regardless of the origin, was *Streptococcus spp*. at 33% and Enterococcus spp. at 30.3% [14]. Other common microorganisms were coagulase-negative *Staphylococci* and *Streptococcus gallolyticus*, but no statistics were mentioned with these statements [15].

In comparison, the most common source of bacteremia that led to TAVR-associated IE was a soft tissue infection or an intravascular source, urological infection, invasive procedures, and dental procedures [19,23,32]. Entry points included gastrointestinal (GI), a femoral access site, an infected knee prosthesis, and an inguinal abscess [22]. Regueiro et al. identified that up to 52.8% of patients developed healthcare-associated IE, and the remainder did not have an identifiable source of bacteremia [32]. Most studies found that the most common microorganism causing IE was *Enterococcus*, and it was usually followed by *S. aureus*, *Streptococcus viridans*, and coagulase-negative *Staphylococci*, especially in the peri-procedural period [15,18,19,22,23,27-29,32]. However, some studies observed *Streptococcus* as the most common microorganism, usually seen in IE stemming from dental procedures [13,21,25,26,29,31]. Two other studies observed that *S. aureus* was the most common microorganism causing IE [29,33]. The risk of *S. aureus* infection was usually healthcare-associated and was increased in skin and soft tissue infections and vascular access [24]. Del Val et al. observed that dental procedures and gastrointestinal infections increased the risk of developed non-*S. aureus* and found that it was the more common, with 63.4% of patients infected [24]. There was also a discrepancy in the microorganisms leading to early IE with *Enterococcus spp.* or *S. aureus* as the two main microorganisms [21,23].

Only one study focused on the origin of SAVR-associated IE being community-acquired. Still, none of the studies analyzing TAVR-associated IE mentioned this occurrence and only mentioned healthcare-acquired IE. More research evaluated the TAVR-associated IE source, which said *Enterococcus *might be the most common organism, but those focusing on SAVR-associated IE found that *S. aureus* was the most common. Lanz et al., on the other hand, concluded that there were no comparable differences between IE causative microorganisms between procedures.

Manifestations

Common manifestations of SAVR-associated IE were not described in any other publications covering this procedure except for the common fever; however, complications were covered in one study [15]. Prosthetic valve vegetations were detected through transesophageal echocardiography (TEE) in up to 70% of patients, even within the ascending aortic graft [16]. Pseudoaneurysms and abscesses were found surrounding the aortic graft and within the aortic annulus in 57% of patients [16]. Moderate to severe aortic regurgitation was also present and occurred more frequently in only prosthetic valves [16]. Further complications, such as septic shock and reduced left ventricular ejection fraction of less than 35%, were also seen in patients [16]. Systemic embolisms also occurred in 20% of patients when viewed via CT or PET scan; however, using other image modalities, systemic embolisms were found in 40.5% of patients [16].

Many studies covered the manifestations of TAVR-associated IE, ranging from symptoms to complications, and most articles found the same results. The most common IE symptom was fever, seen on admissions, and other symptoms, such as chills, rigor, sweats, lethargy, malaise, and acute heart failure [19,23-25,32]. The majority of patients experienced complications during hospitalization, such as vegetation, regurgitations, abscess, neurological symptoms, systemic embolisms, renal failure, and uncontrolled infections, causing bacteremia and leading to severe sepsis and septic shock [18,19,21-23,25,27,29,34]. Vegetations occurred frequently and typically developed on the prosthetic leaflet with an average size of 10.7 mm [19,23,32]. Bjursten et al. observed that 21% of cases had vegetation that developed on the mitral valve, and Brouwer et al. found similar results in 25% [21,22]. Olsen et al. noted that in their study, up to 44.4% of patients had negative TEE results [27]. Also needs to be considered is the paravalvular extension of the infection, which also leads to aortic regurgitation [19]. Developed abscesses were also observed, typically involving the valve's periannular region [32]. However, patients who developed in-hospital IE were found to have increased rates of septic shock, acute kidney injury, cardiogenic shock, bleeding requiring transfusion, and myocardial infarction [31].

Del Val et al. observed specific manifestations in patients whose IE developed from an *S. aureus* infection. They observed that* S. aureus*-infected patients were less likely to develop vegetation and presented with at least one complication, such as systemic embolism, neurological manifestations, new onset heart failure, acute renal failure, bacteremia, and septic shock [24]. In a later study, Del Val et al. also observed that acute renal failure complications had significantly decreased after 2013, regardless of the etiological origin [24]. Regueiro et al. also observed a difference in manifestations between self-expanding and balloon-expanding valves. They observed that patients with self-expanding valves had higher rates of moderate to severe paravalvular aortic regurgitation, periannular complications, major or life-threatening bleeding, and acute kidney injury than balloon-expanding valves [34]. Patients with balloon-expanding valves had higher stroke rates and systemic embolisms [34]. Both valves developed vegetations, but vegetations at the stent frame were seen more in the self-expanding valve group, and vegetations at the valve leaflets were seen more in the balloon-expanding valve group [34].

In comparing the IE manifestations between each procedure, TAVR patients tended to have less frequent fevers than SAVR [15]. Calderon-Parra et al. also observed no other differences in clinical manifestations between procedures; however, Lanz et al. observed that SAVR-associated IE patients were more likely to have abscess formation than TAVR-associated IE [14,15]. Lanz et al. [14] also saw one similarity in Calderon-Parra et al.’s paper. There was no significant difference in the incidence of strokes in patients who developed IE following both procedures [14]. Also, comparing previously mentioned articles in this section, it does appear that SAVR has a higher prevalence of vegetation formation overall.

Treatment

Articles covering general AVR-associated IE generally did not mention treatment methods, but procedure-specific articles did. In SAVR-associated IE, only one study describes treatment methods. Most non-surgical patients received six weeks of antibiotic treatment [16]. Ceftriaxone and gentamicin were the main antibiotics used, but 65% of patients had persistent signs of infections and qualified for surgery [16]. Of those, only 62% of patients received surgery for post-graft complications, possibly due to risk factors in the patients who did not undergo surgical treatment [16].

Regarding TAVR-associated IE, significantly more research evaluated different aspects of treatment, including the duration of hospital stay. The median hospital duration varied from an average of eight days to a median of 31.2 days; however, hospital stays decreased after 2013 [22,23,26]. Most patients were treated with antibiotics, even when surgery indications were present in many patients [23,32]. The most common antibiotics used were vancomycin, especially for *S. aureus*-infected patients, gentamicin, and beta-lactams [19,23,24]. Brouwer et al. observed that in their study, all patients were treated conservatively with antibiotics, and none of the patients underwent re-intervention of their TAVR prosthesis, which was also seen in Kolte et al.’s study [18,22].

When indications for surgery were met, only a tiny percentage of patients underwent surgical intervention in patients with TAVR-associated IE [19]. A percentage (21%) of* S. aureus*-infected patients underwent surgical treatment, but management rates were similar to non-*S. aureus*-infected patients [24]. Open-heart surgery with AVR, TAVR explantation, redo TAVR, and pacemaker extraction were common surgical treatments, with in-hospital IE having increased rates of permanent removal of a pacemaker [18,21,23,31]. Patients sometimes require implantation of a pacemaker following IE, and patients with self-expanding valves were more likely to receive them than those with balloon-expanding valves [34]. Patients with a redo-TAVR tended to have an endovascular TAVR compared to transapical TAVR [18].

One study compared the median number of days a patient was in hospital for SAVR- and TAVR-associated IE and found that the duration in TAVR-associated IE was 398 compared to SAVR-associated IE, which was 469, but significance was not assessed [35]. SAVR- and TAVR-associated IE had a similar prevalence of surgical indications, but TAVR patients tended to be treated more conservatively with antibiotics, as previously mentioned. This could be due to the same risk factors that lead to the need for a TAVR to be performed.

Outcomes

In assessing general AVR-associated IE, it was observed that prosthetic valve endocarditis was associated strongly with all-cause mortality following diagnosis [13]. For patients diagnosed with IE, the all-cause mortality was as high as 42.3% following a year after surgery [14].

Studies focusing on SAVR-associated IE observed that the survival rate in patients with coronary valve grafts was 31.25% compared to 4.76% in patients who received supracoronary aortic grafts [16]. Medranda et al. observed that 27.3% of patients who had undergone surgical intervention survived [25]. Patients who did not undergo surgery had an in-hospital mortality rate of 7.1% [16]. The in-hospital mortality rate was 14%, but there were higher rates in prosthetic valves only compared to prosthetic valves with ascending aortic grafts [12,16]. Patients were more at risk for any cause of death and cardiovascular death at one year, and all-cause mortality had an average of 50.4% [12-14,18].

The survival rates of patients with TAVR-associated IE at discharge were 61.4% and 54.4% at one year, and 37.5% of patients survived an average of 709 days following conservative management [19,33]. Non-community onset, late-onset, *S. aureus*, and abscess formation were significantly associated with worse outcomes [21]. Significant independent factors associated with in-hospital mortality were *S. aureus* etiology, higher risk determined by the logistic EuroSCORE, septic shock, cardiogenic shock, and IE-related complications, such as new onset heart failure, acute renal failure, and stroke during hospitalization [23,32]. Other factors found to affect the overall mortality included periannular complications, heart failure, non-streptococcal/enterococcal etiology, being female, diabetes mellitus, lack of surgical management, persistent bacteremia, septic shock, and chronic obstructive pulmonary disease (COPD) history [24,28]. Patients with in-hospital IE were found to have increased death rates and had a significant seven-fold risk for all-cause mortality [29]. Patients with IE had a three-fold higher mortality risk after accounting for procedural complications and comorbidities than patients who did not develop IE [26]. Mortality was higher in non-operated patients than those who received surgical treatment, which supports some concern about primarily using conservative methods for treatment [23].

The in-hospital mortality range had an average of 25.7%, and patients infected with *S. aureus* had a higher in-hospital mortality rate of 47.8% [12,18,21,23,24,27,28,32]. Del Val et al. observed that in-hospital mortality had declined since 2013, possibly due to the advancement of treatment procedures [23]. One-year mortality rates ranged from an average of 39.5% [12,14,21,23,28]. Only Bjursten et al. observed the five-year mortality and assessed it as 28.9% [21]. Mortality averaged 31% overall once IE was diagnosed [13,22,27,30,33]. Regarding early, intermediate, and late-onset IE, the mortality rate was 25%, 25%, and 100%, respectively [33]. Del Val et al. found that patients infected with *S. aureus* had a higher late mortality rate of 71.5% [24]. Evaluating the different types of valves, there was an insignificant difference in in-hospital death between self-expandable and balloon-expandable valves [32]. Moreover, the rate at which patients who survived their diagnosis developed a second episode was 9.4% [32].

Comparing both procedures, strokes occurred in most patients two years following treatments for both SAVR- and TAVR-associated IE [14]. A portion (18.9%) of patients with TAVR-associated IE died compared to 14.1% of SAVR-associated IE [35]. The adjusted all-cause death rate in TAVR-associated IE was significantly higher immediately after diagnosis than in SAVR-associated IE [35]. In-hospital and one-year mortality were similar; however, in patients with surgical indications, those with TAVR-associated IE had a higher in-hospital mortality [14,15]. This was confirmed by Moriyama et al., who observed that the surgical treatment of IE was independently associated with a reduction in in-hospital mortality [20].

Some limitations of this study were the limited research on SAVR-associated IE and the fact that demographic variations concerning countries were not assessed. The reduced amount of research on SAVR-associated IE could be due to the shift in TAVR procedures; however, SAVR is still necessary for some patients with certain risk factors. Demographics were not assessed as there was only one article per region for the most part, as well as varying information between articles as some excluded symptoms and treatments, among others. Future studies may want to consider assessing area and ethnic variations in the development of IE in TAVR-associated and the types of prosthetic valves.

Table 1 displays information on each article used to gather the information used in this paper.

**Table 1:** Summary of the articles used in this study per Preferred Reporting Items for Systematic Reviews and Meta-Analyses (PRISMA). TAVR: transcatheter aortic valve replacement, SAVR: surgical aortic valve replacement, IE: infective endocarditis, PVE: prosthetic valve endocarditis, AAG: ascending aortic graft, TAVI: transcatheter aortic valve implantation, SEV: self-expandable valve, BEV: balloon-expandable valve, COPD: chronic obstructive pulmonary disease, DM: diabetes mellitus, eGFR: estimated glomerular filtration rate, CC: contemporary cohort

	Author	Country	Design and study population	Findings	Conclusion
1	Lanz et al., 2021 [14]	Switzerland	Experimental study (n = 4301)	A portion (0.5%) of patients who underwent TAVR and 1.1% who underwent SAVR developed IE. Patients who developed IE tend to have diabetes compared to those without. The all-cause mortality rate among patients with IE was 27.3% in the TAVR group and 51.8% in the SAVR group.	There was an overall low incidence of IE regardless of the surgery; however, there was a lower incidence in the TAVR group. However, once IE is present, the mortality is relatively high.
2	Medranda et al., 2021 [25]	USA	Retrospective cohort study (n = 396)	A portion (2.8%) of low-risk patients developed PVE at a median day of 379 following TAVR. *Streptococcus* was the most common organism found to infect the prosthetic valve at 36.3%. Early PVE occurring in <365 days happened in 54.5% of patients; 50% showed evidence of an embolic stroke.	PVE has a low incidence following TAVR in low-risk patients but was associated with substantial mortality and morbidity, with stroke frequently leading to a worse outcome.
3	Mentias et al., 2020 [26]	USA	Retrospective cohort study (n = 134,717)	A portion (1.39%) of patients developed endocarditis following TAVR. The most common organism involved with *Staphylococcus* at 22%. Crucial predictors for IE were male sex, younger age at TAVR, end-stage renal disease, prior endocarditis, and repeat TAVR. Thirty-day mortality was 18.5%, and one-year mortality was 45.6%.	IE of TAVR is low and currently declining, but once diagnosed, it is associated with a bad prognosis, with almost half of the patients passing within one year.
4	Olsen et al., 2015 [27]	Denmark	Retrospective cohort study (n = 509)	A portion (3.5%) of patients were diagnosed with PVE and were most frequently diagnosed in the first year following implantation. Ninety-four percent of patients were conservatively treated. 22% of patients died from IE or complications from treatment.	IE following TAVI had a higher incidence than those who underwent surgically implanted valves. There was an increased risk in those with vascular complications and suboptimal valve deployment.
5	Kolte et al., 2018 [18]	USA	Retrospective cohort study (n = 95,383)	The incidence rates of IE after TAVI and SAVR from the Nationwide Readmissions Databases were 1.7% and 2.5% per person-year, respectively. Incidence rates and the average number of days a patient acquired IE after TAVI or SAVR were similar. Common causes of IE post-TAVI are *Staphylococcus, Streptococcus,* and *Enterococcus.*	The incidence rate of early IE in a nationally represented cohort of TAVI patients was 1.7% per person-year. Readmission of post-TAVI patients noted that age, invasive produces, complications, and comorbidities were associated with the incidence of IE.
6	Kaur et al., 2022 [33]	USA	Retrospective cohort study (n = 494)	The IE registry showed that 2% of patients within 13 years had confirmed TAVR-associated IE. Most of these cases were of intermediate onset, caused by *Staphylococcus aureus*, at an average age of 78.1 years old. IE mortality rate was 40%, 25% in cases with early and intermediate onset, and 100% in patients with late beginning of IE.	Of the confirmed TAVR-IE cases in this study, *Staphylococcus aureus* was the primary causative agent, and most were of intermediate onset.
7	Glaser et al., 2017 [17]	Sweden	Retrospective cohort study (n = 26,580)	A percentage of 3.5% were hospitalized for infective endocarditis. The incidence rate for PVE was 0.57% per person-year, and most of these cases pertained to patients with bioprosthetic valves.	Bioprosthetic surgical aortic valve replacement was noted to correlate more with post-implantation infective endocarditis.
8	Garcia-Arribas et al., 2021 [16]	Spain	Retrospective cohort study (n = 1,654)	Some patients (91.9%) ofwith aortic grafts developed IE. *Staphylococci* caused 32% of patients with IE, while *Streptococci viridans* mainly caused patients with composite valve grafts. The mortality rates of patients who received a composite valve graft versus a supracoronary graft were 4.8% and 31.3%, respectively.	Mortality was not higher in patients with prosthetic aortic valve IE and AAG than in those with prosthetic IE. Imaging can play a role in diagnosing IE patients and determining whether the patient qualifies for surgery or can be managed by antibiotic therapy.
9	Fauchier et al., 2020 [35]	France	Retrospective cohort study (n = 107,806)	The incidence rates of acquiring IE following TAVI and SAVR patients were 1.89 and 1.40 events per 100 person-years, respectively. The mortality rate was higher in patients who acquired IE post-TAVI versus post-SAVR at 43% and 32.8%, respectively.	There is a similar risk of acquiring IE between TAVI and SAVR patients. Patients diagnosed with IE after receiving TAVI were noted to have a higher mortality rate.
10	Del Val et al., 2022 [24]	Canada	Retrospective study (n = 573)	A portion (24.6%) of patients post-TAVR were identified to have *Staphylococcus aureus *IE, with self-expanding valves having an increased likelihood of early IE. Factors including sepsis, neurologic symptoms, major bleeding, systemic embolism, and IE-associated cardiac device implant increased the risk of *Staphylococcus aureus * IE to 84.6% if multiple were involved. The in-hospital and 2-year mortality rates for patients with SA IE were 47.8% and 71.5%, respectively.	*Staphylococcus aureus*-related IE after TAVR was found in 25% of patients with high in-hospital and late mortality rates. Specific factors also proved an increased likelihood of *Staphylococcus aureus *IE. Surgery should be considered for further patient management due to improved *Staphylococcus aureus *IE outcomes.
11	Yeo et al., 2018 [31]	USA	Cross-sectional Study (n = 41, 025)	A portion (0.3%) of patients who underwent TAVR were found to have developed in-hospital IE. 20.8% of post-TAVR in-hospital IE was caused by *Streptococcus viridians*, followed by *Staphylococcus aureus* (16.7%) and *Enterococci* (8.3%). These patients were noted to have increased rates of septic shock (16.7%), death (20.8%), cardiogenic shock (12.5%), bleeding requiring transfusion (29.2%), myocardial infarction (12.5%), acute kidney injury requiring hemodialysis (16.7%), and removal of a permanent pacemaker (4.2%). Post-TAVR in-hospital IE risk factors are drug abuse, HIV, and a younger age.	Following TAVR, IE developed within the same hospital admission in 0.3% of the patients. In these patients, greater rates of mortality and adverse outcomes were noted. Risk factors, such as a history of drug abuse, younger age, or HIV infection, placed patients at an increased risk of developing IE in the hospital post-TAVR.
12	Stortecky et al., 2020 [29]	Switzerland	Case-control study (n = 7,203)	Of the 7,203 patients who underwent TAVR, 149 developed IE with respective incidences of 2.59, 0.71, and 0.40 events per 100-person years for peri-procedural, delayed-early, and late endocarditis, respectively. The *Enterococcus* species was the most common microorganism in patients with early endocarditis. IE post-TAVR was associated with a lack of pre-dilatation, a younger age, and treatment in a catheterization laboratory. The patients who developed IE were at an increased risk of stroke and mortality.	The development of IE after TAVR commonly develops in the earlier periods and is typically caused by the *Enterococcus* species. Development of IE post-TAVR was found to be associated with risks of stroke and death.
13	Rodriguez-Vidigal et al., 2019 [28]	Spain	Observational study (n = 200)	Out of the 120 patients who received TAVI, 11 patients were found to have developed IE. 36.4% was the in-hospital mortality rate, while the median number of days for IE to develop from the TAVI procedure was 112. The patients who developed IE were found to have a greater EuroSCORE, greater frequency of neoplasia history, and were younger. The identified organisms were *Enterococcus faecalis* and coagulase-negative *Staphylococcus. *	A greater incidence of IE post-TAVI was found in this study when compared to the previously conducted multicenter series. IE post-TAVI was concluded to result in higher mortality and is linked with a worse baseline situation.
14	Regueiro et al., 2019 [34]	UK	Retrospective cohort study (n = 6363)	Of those who developed IE following TAVR, 47% had a self-expandable valve (SEV), while 53% had a balloon-expandable valve (BEV). The time between the TAVR procedure and the development of IE was found to be 5.5 and 5.3 months for SEV and BEV, respectively. IE caused by *Enterococci* was found to be more common in patients with an SEV (18.6%). The location of vegetation varied between the stent frame and the valve leaflet. Death in-hospital rates were 35.6% and 37.7% for SEV and BEV, respectively.	It was concluded that the development of IE post-TAVR had varying microorganisms, embolic complications, and vegetation locations based on the type of valve used (balloon-expandable valve vs. self-expanding valve).
15	Summers et al., 2019 [13]	US	Cohort study (n = 8,530)	PVE had an overall incidence rate of 5.06 per 1,000 person-years. There was no difference in the cumulative incidence of TAVR-PVE and SAVR-PVE. There was no significant difference in the timing of infection after AVR between TAVR-PVE and SAVE-PVE. The occurrence of PVE was linked to pre-existing cirrhosis, pulmonary disease, and renal insufficiency. *Staphylococcus* infection occurred more frequently after SAVR vs TAVR. PVE was associated with overall mortality following the diagnosis of endocarditis.	The occurrence of PVE remains low in modern AVR practice, regardless of whether replacement is done through surgical or transcatheter approaches. No variation exists in terms of PVE incidence, predictors, or risk between TAVR and SAVR.
16	Calderon-Parra et al., 2022 [15]	Spain	Observational study (n = 633)	A portion (1.41%) of patients who underwent SAVR and 4.65% who underwent TAVI developed IE (p = 0.016). TAVI patients were found to develop early IE more frequently (p = 0.006). In a propensity score analysis, the risk of IE did not differ between groups. Risk factors associated with IE following TAVI were younger age, COPD, complicated DM, peripheral arteriopathy, and advanced heart failure.	The risk attributable to each group was similar following a propensity score analysis; however, early IE was significantly higher in patients who underwent TAVI.
17	Regueiro et al., 2016 [32]	Canada	Retrospective cohort study (n = 20006)	The incidence of IE after TAVR was 1.1% per person-year, with a median age of 80. The median time of developing IE following TAVR was 5.3 months. Factors associated with a higher risk of developing IE were a younger age, male sex, diabetes mellitus, and moderate-severe aortic regurgitation. *S. aureus *and *Enterococci *species were the most frequent organisms that led to IE. Surgery was performed in 14.8% of patients, and the in-hospital mortality rate was 36%. In-hospital mortality was associated with heart failure, higher logistic EuroSCORE, and acute kidney injury.	Patients who developed IE following TAVR were at significant risk if they were younger, had a history of diabetes mellitus, were male, and had moderate-severe residual aortic regurgitation. The patients who developed IE had high rates of in-hospital and 2-year mortality.
18	Tabata et al., 2020 [30]	Germany	Prospective cohort study (n = 1448)	A portion (1.2%) of TAVI patients developed IE during a follow-up of 294 days. Age and residual paravalvular leakage >1 following TAVI are the main predictors for IE occurrence. IE following TAVI was an independent predictor of long-term mortality.	Age and residual leakage were predictors of IE following TAVI, and IE was associated with long-term mortality.
19	Moriyama et al., 2019 [20]	Japan	Retrospective study (n = 6463)	The incidence of IE following TAVR was 3.4/1000 person-years and 2.9/1000 person-years following SAVR. There was no significant difference between TAVR and SAVR concerning risks. Males and patients with vascular access-site infection or deep sternal wound infections were associated positively with IE, but the procedure was not associated. The mortality rate was 37% for IE one month following surgery and was increased to 52.5% at one year.	The incidences of IE between SAVR and TAVR were similar, as well as risk factors. Males and patients with vascular access-site infection or deep sternal wound infections were associated positively with IE.
20	Bjursten et al., 2019 [21]	Sweden	Retrospective study (n = 4336)	A portion (2.38%) of individuals experienced one episode of PVE. The incidence of PVE following a TAVI was 1.42% for the first year, 0.80% for 1-5 years and 0.52% for 5-10 years. Fifty-one patients had an early PVE, and 52 had a late PVE. S. aureus was more common in early PVE (<1 year). In-hospital mortality was 16.8%. Infection with S. aureus, root abscess, non-community acquisition, and late PVE were associated with a higher 6-month mortality. One-year survival after PVE diagnosis was 58%, and 5-year survival was 29%.	The incidence of IE decreases progressively each year. High body surface area, reduced eGFR, and renal function emerged as significant predictors of PVE, and there was a notable rise in the prevalence of renal dysfunction with advancing age in the TAVI patient population. *S. aureus* was associated with both fatal outcomes and early IE.
21	Brouwer et al., 2020 [22]	Netherlands	Retrospective study (n = 3968)	Sixteen patients developed an episode of PVE after TAVR during a median follow-up period of 33.5 months with an incidence rate of 0.14 events per 1000 person-years. The overall incidence was 0.4%, with a higher incidence of early PVE. The mortality within the hospital setting was 25%, with an overall mortality rate of 31%. The most associated pathogen was *Enterococcus faecalis*. Baseline characteristics between the PVE and control groups differed significantly. The median age was 81.5 years old, with over half of the patients male in the PVE group versus the control group, 82.1 years old.	PVE post-TAVR was associated with a high in-hospital mortality rate compared to previous nationwide studies. All patients were managed conservatively, receiving solely intravenous antibiotics, and none required re-intervention. Aortic valve regurgitation developed in 50% (8/16) of the patients.
22	Butt et al., 2019 [12]	Denmark	Observational cohort study (n = 6,409)	A portion (4.4%) of patients with TAVR and 4.9% with SAVR were admitted with IE during a mean follow-up for 3.6 years. The crude incidence rates of IE were 1.6 and 1.2 events per 100 person-years in TAVR and SAVR patients, respectively. The cumulative 5-year risk of IE was 5.8% in TAVR and 5.1% in SAVR.	The incidence rate of IE within 5 years after TAVR was 5.8%, and this rate did not display a statistically significant difference when compared to the incidence observed after SAVR.
23	Cahill et al., 2022 [19]	United Kingdom	Retrospective cohort study (n = 107,976)	Among patients who underwent TAVR, 0.98% experienced the development of IE during a median follow-up of 23.8 months, resulting in an overall incidence of 3.64 per 1000 person-years. In parallel, among those who underwent SAVR, 2.24% encountered IE over a median follow-up of 53.8 months with an overall incidence of 4.82 per 1000 person-years. The cumulative incidence of IE at 60 months was significantly greater following SAVR than TAVR. Survival rates for TAVR-IE stood at 54.4% in one year, with unfavorable outcomes linked to occurrences of shock or stroke. *Enterococci *was the most common cause of TAVR-IE.	The incidence of IE after TAVR is lower than that after SAVR. The incidence of IE showed a marked increase in males, individuals who received mechanically expandable and balloon-expandable valves, and those with a higher post-procedural peak gradient.
24	Del Val et al., 2021 [23]	International	Observational study (n = 552)	A total of 285 patients with IE after TAVR were included in the historical cohort (HC; June 2005 to December 2013) and 263 patients in the contemporary cohort (CC; January 2014 to May 2020). The incident rates of IE were comparable in both groups, but early IE cases were significantly lower in the CC. *Enterococci *were the predominant microorganisms identified. While most patients had complicated surgical interventions, rates remained low. In-hospital mortality was lower in the CC.	Over time, there has been a decreasing rate of in-hospital and one-year mortality. Patients in the CC presented a lower surgical risk profile, indicating an improved clinical condition.

## Conclusions

IE is a life-threatening and rare complication that can occur following AVR, whether it is SAVR or TAVR. AVR is usually performed to correct aortic stenosis from old age or patients with congenital bicuspid valves to improve LVOT. SAVR involves open-heart surgery, where the damaged valve is excised and replaced with a prosthetic valve or graft to establish an adequate blood flow. TAVR is a minimally invasive procedure, implanting a prosthetic valve through a catheter-based approach. This technique evades the need for open heart surgery and is an optimal alternative for most patients who require AVR. TAVR has tremendous benefits for patients in need and is considered much safer than SAVR, but not all patients can undergo TAVR.

There were no significant differences between SAVR- and TAVR-associated IE concerning incidence, risk factors, and mortality rates. Some etiological differences can account for specific symptoms, such as an increased rate of abscess formation in patients with SAVR-associated IE, as *S. aureus *is a common abscess-forming microorganism. Treatments also varied as patients with SAVR-associated IE were more likely to undergo surgery when indicated, and in-hospital mortality rates were considerably low; this could be due to certain risk factors that led to surgical interventions being more favorable. AVR is a needed operation with no pharmacological substitutes providing the equivalent relief. Because IE incidences and mortality rates are similar between each surgery, research should focus on ways to prevent IE from occurring, as both procedures are done out of necessity. However, a few TAVR studies compared the incidence and manifestations of IE in patients who received balloon-expandable and self-expanding valves. Balloon-expanding valves have increased the incidence of IE, and vegetation locations are explicitly different between each valve. As the shift to move more patients to receive TAVR as the procedure tactics advance, it would be essential to compare and evolve placement tactics and prosthetic valves to reduce and prevent IE from occurring as much as possible.
